# Evolutionary plasticity of cyanobacteria under persistent anoxia: mechanistic insights from marine blue holes and global ecological implications

**DOI:** 10.1128/aem.00251-26

**Published:** 2026-05-18

**Authors:** Hanrong Qiu, Zhenyan Zhang, Haifeng Qian

**Affiliations:** 1 College of Environment, Zhejiang University of Technology 12624, Hangzhou, People's Republic of China; 2 Institute for Advanced Study, Shaoxing University 66326, Shaoxing, People's Republic of China; Michigan State University, East Lansing, Michigan, USA

**Keywords:** cyanobacteria, oxygen gradients, evolutionary plasticity

## Abstract

Cyanobacteria are generally viewed as obligate oxic photoautotrophs. However, this paradigm was challenged by Z. Li, H. Zhang, T. Wei, L. He, and Y. Wang in *Applied and Environmental Microbiology*(92:e02576-25, 2026, https://doi.org/10.1128/aem.02576-25); this group identified transcriptionally active *Synechococcus* in the dark, permanently anoxic Yongle Blue Hole using integrated metagenomic and transcriptomic analyses. This finding suggests adaptive streamlining under long-term oxygen limitation, expands the recognized ecological range of phototrophic microorganisms, and highlights the potential relevance of microbial adaptation to future ocean deoxygenation.

## COMMENTARY

Recent studies of microbial dormancy, functional redundancy, and community regulation further emphasize the capacity of microbial communities to reorganize in response to environmental stress (1, 2). Rather than being passive responders to environmental change, microorganisms have long shaped Earth’s biogeochemical history. A defining example is the Great Oxidation Event approximately 2.4 billion years ago, when oxygenic photosynthesis by cyanobacteria fundamentally altered planetary redox conditions and global biogeochemical cycles (3). Understanding how foundational lineages such as cyanobacteria respondto and restructure the changing conditions is therefore important for anticipating the future trajectory of ocean biogeochemistry.

For decades, cyanobacteria have been regarded as obligate photoautotrophs whose ecological distribution is constrained by light availability and oxic conditions. This view is challenged by the recent report of transcriptionally active *Synechococcus* populations in the permanently dark and anoxic deep waters of the Yongle Blue Hole in the South China Sea. Phylogenetically, these lineages cluster near certain sponge-associated cyanobacteria, yet they retain core photosynthetic and carbon fixation modules while showing reduced capacities for oxidative stress defense, osmotic adaptation, and circadian regulation. Notably, photosynthesis- and aerobic respiration-related genes remain genomically present but are largely transcriptionally silent under *in situ* anoxic conditions. Rather than indicating simple genome degeneration, these findings suggest a specialized adaptive state shaped by long-term environmental stability and stress.

As ocean conditions continue to change, the adaptive responses of microorganisms may have important consequences for future biogeochemical cycling. Cyanobacteria once transformed the planet through oxygenic photosynthesis, and lineages adapted to persistent anoxia may likewise influence microbial ecosystem function under ongoing ocean deoxygenation (4, 5). In this context, the Yongle Blue Hole finding is important not only because it expands the known ecological range of cyanobacteria but also because it provides insight into how phototrophic microorganisms may persist under long-term oxygen limitation.

**Fig 1:**
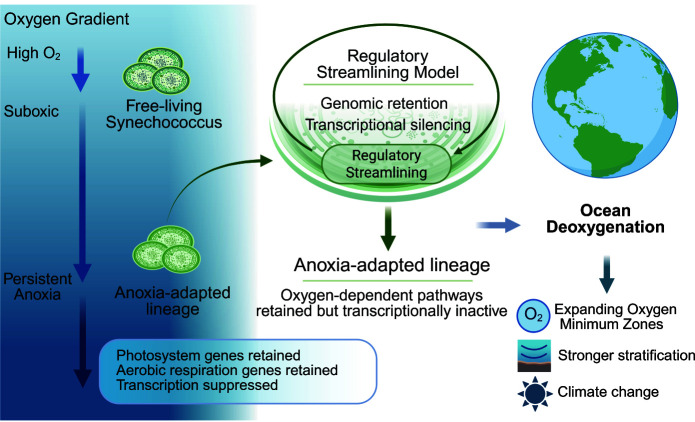
Conceptual model of cyanobacterial adaptation to persistent anoxia in the Yongle Blue Hole. Anoxia-adapted *Synechococcus* retains core metabolic potential while showing reduced regulatory and stress-response capacities, suggesting regulatory streamlining under long-term oxygen limitation (8).

From a mechanistic perspective, the blue hole cyanobacteria exhibit a striking combination of traits: core photosynthetic modules are retained, whereas regulatory and defensive systems typically associated with oxic lifestyles are reduced or absent (Fig. 1). This pattern suggests that under prolonged and stable environmental stress, evolution may favor the preservation of core metabolic capacity while reducing regulatory complexity and maintenance costs. Comparable strategies have been observed in other microorganisms adapted to low-oxygen environments, including bacteria that combine respiratory and fermentative processes to reduce oxygen dependence (6). Viewed in this light, the published data are consistent with a process of regulatory streamlining, in which long-term residence in a stable but extreme habitat selects for selective simplification rather than complete metabolic loss. Thisinterpretation also resonates conceptually with the adaptive gene-loss logic described by the Black Queen hypothesis (7).

Importantly, such adaptation is unlikely to be restricted to rare extreme habitats alone. In the ocean, oxygen is more often distributed as a continuum than as a simple oxic–anoxic divide (9). This suggests that microbial adaptation may proceed through intermediate stages, with gradual ecological differentiation emerging along persistent oxygen gradients. From this perspective, the anoxia-adapted cyanobacteria identified in deep blue holes may represent one end of a broader adaptive spectrum rather than an isolated anomaly. A key question, therefore, is whether cyanobacterial lineages across the wider ocean also display progressive ecological shifts and evolutionary trajectories in response to declining oxygen availability (10)?

More broadly, the Yongle Blue Hole finding can be viewed in the context of ongoing global ocean deoxygenation. As dissolved oxygen declines, oxygen minimum zones expand, and stratification intensifies, persistently low-oxygen habitats are becoming more widespread in the ocean (11, 12). In such settings, lineages once restricted to localized anoxic environments may acquire broader ecological importance. If cyanobacteria are able to persist through regulatory streamlining and metabolic reorganization under prolonged oxygen limitation, comparable adaptive tendencies may also be relevant to other phototrophic microorganisms. Prochlorococcus, for example, already exhibits marked genome reduction and diversification across environmental gradients (13), while other phototrophs retain substantial metabolic flexibility (14). Although the specific trajectories will likely differ among lineages, the Yongle Blue Hole discovery encourages a broader reevaluation of how phototrophic microorganisms may respond to a progressively deoxygenated ocean.

A central uncertainty, however, is whether microbial adaptation can keep pace with the current rate of anthropogenic change. Coastal eutrophication is expanding hypoxic zones; climate warming is strengthening ocean stratification; and acidification is continuing across marine systems (15). Compared with environmental change over geological timescales, these shifts are unfolding much more rapidly. If microbial populations can adjust through regulatory streamlining and metabolic reconfiguration, they may continue to influence biogeochemical cycling under emerging ocean conditions (16). If not, substantial community restructuring or functional loss may follow. How microorganisms will respond to the combined pressures of deoxygenation, warming, and acidification remains an open question, with important implications for future marine ecosystem function (17).

However, our understanding of microbial life in extreme marine environments remains limited. In this respect, the Yongle Blue Hole discovery is especially informative: it extends the recognized ecological boundaries of phototrophic microorganisms and suggests that deep and persistently oxygen-depleted systems may harbor a broader diversity of uncharacterized lineages than currently appreciated. Continued exploration of such environments should help refine existing views of microbial adaptation and may also improve our ability to anticipate ecological responses to future ocean change.

Future work should now focus on linking environmental observations with targeted validation. In particular, isolation or enrichment of anoxia-adapted cyanobacteria would help clarify whether their retained photosynthetic machinery remains reversible and to what extent core metabolic functions persist under prolonged anoxia. Experimental evolution under controlled oxygen gradients may likewise help test whether comparable patterns of regulatory simplification emerge in other cyanobacterial lineages.

In summary, the Yongle Blue Hole cyanobacteria broaden the known ecological range of phototrophic microorganisms and provide a useful example of how long-term oxygen limitation may reshape microbial regulatory organization without necessarily eliminating core metabolic capacity.

The discovery of anoxia-adapted cyanobacteria in the Yongle Blue Hole expands the recognized ecological boundaries of phototrophic microorganisms and offers new insight into how cyanobacterial lineages may persist under long-term oxygen limitation. Rather than simply reflecting metabolic loss, the published evidence supports a pattern of selective regulatory simplification coupled with retention of core metabolic potential. In the context of ongoing ocean deoxygenation, this finding raises broader questions about how phototrophic microorganisms may adapt across oxygen gradients and how such adaptations may ultimately influence future marine ecosystem function and biogeochemical cycling.

## References

[1] Zhang Z, Zhang Q, Chen B, Yu Y, Wang T, Xu N, Fan X, Penuelas J, Fu Z, Deng Y. 2024. Global biogeography of microbes driving ocean ecological status under climate change. Nat Commun 15:4657. doi:10.1038/s41467-024-49124-0 38822036 PMC11143227

[2] Bradley JA. 2025. Microbial dormancy as an ecological and biogeochemical regulator on Earth. Nat Commun 16:3909. doi:10.1038/s41467-025-59167-6 40280922 PMC12032139

[3] Sánchez-Baracaldo P, Bianchini G, Wilson JD, Knoll AH. 2022. Cyanobacteria and biogeochemical cycles through Earth history. Trends Microbiol 30:143–157. doi:10.1016/j.tim.2021.05.008 34229911

[4] Chen Q, Tang K, Zhai W, Zhu Z, Terence Yang J-Y, He Z, Li M, Kao S-J, Yang J, Zheng Q, et al.. 2025. Microbial responses to ocean deoxygenation: revisiting the impacts on organic carbon cycling. iScience 28:112826. doi:10.1016/j.isci.2025.112826 40655090 PMC12246640

[5] Sun G, Zou Q, Wang B. 2025. The interplay of carbon and nitrogen cycling driven by watershed microorganisms. Front Microbiol 16:1696238. doi:10.3389/fmicb.2025.1696238 41640408 PMC12866615

[6] Trojan D, García-Robledo E, Hausmann B, Revsbech NP, Woebken D, Eichorst SA. 2024. A respiro-fermentative strategy to survive nanoxia in Acidobacterium capsulatum. FEMS Microbiol Ecol 100:fiae152. doi:10.1093/femsec/fiae152 39557655 PMC11636273

[7] Morris JJ, Lenski RE, Zinser ER. 2012. The black queen hypothesis: evolution of dependencies through adaptive gene loss. mBio 3:e00036-12. doi:10.1128/mBio.00036-12 22448042 PMC3315703

[8] Li Z, Zhang H, Wei T, He L, Wang Y. 2026. Anoxia-adapted cyanobacteria in a marine blue hole. Appl Environ Microbiol 92:e02576-25. doi:10.1128/aem.02576-25 41728996 PMC12997750

[9] Wright JJ, Konwar KM, Hallam SJ. 2012. Microbial ecology of expanding oxygen minimum zones. Nat Rev Microbiol 10:381–394. doi:10.1038/nrmicro2778 22580367

[10] Shih PM, Hemp J, Ward LM, Matzke NJ, Fischer WW. 2017. Crown group oxyphotobacteria postdate the rise of oxygen. Geobiology 15:19–29. doi:10.1111/gbi.12200 27392323

[11] Breitburg D, Levin LA, Oschlies A, Grégoire M, Chavez FP, Conley DJ, Garçon V, Gilbert D, Gutiérrez D, Isensee K, et al.. 2018. Declining oxygen in the global ocean and coastal waters. Science 359:eaam7240. doi:10.1126/science.aam7240 29301986

[12] Schmidtko S, Stramma L, Visbeck M. 2017. Decline in global oceanic oxygen content during the past five decades. Nature 542:335–339. doi:10.1038/nature21399 28202958

[13] Biller SJ, Berube PM, Lindell D, Chisholm SW. 2015. Prochlorococcus: the structure and function of collective diversity. Nat Rev Microbiol 13:13–27. doi:10.1038/nrmicro3378 25435307

[14] Stoecker DK, Hansen PJ, Caron DA, Mitra A. 2017. Mixotrophy in the marine plankton. Annu Rev Mar Sci 9:311–335. doi:10.1146/annurev-marine-010816-060617 27483121

[15] Altieri AH, Gedan KB. 2015. Climate change and dead zones. Glob Chang Biol 21:1395–1406. doi:10.1111/gcb.12754 25385668

[16] Louca S, Polz MF, Mazel F, Albright MBN, Huber JA, O’Connor MI, Ackermann M, Hahn AS, Srivastava DS, Crowe SA, et al.. 2018. Function and functional redundancy in microbial systems. Nat Ecol Evol 2:936–943. doi:10.1038/s41559-018-0519-1 29662222

[17] Boyd PW, Cornwall CE, Davison A, Doney SC, Fourquez M, Hurd CL, Lima ID, McMinn A. 2016. Biological responses to environmental heterogeneity under future ocean conditions. Glob Chang Biol 22:2633–2650. doi:10.1111/gcb.13287 27111095

